# Impact of COVID-19 prevention and control on tuberculosis and scarlet fever in China’s Guizhou

**DOI:** 10.1038/s41598-023-36263-5

**Published:** 2023-06-12

**Authors:** Jian Zhou, Hui-Juan Chen, Ting-Jia Lu, Pu Chen, Yan Zhuang, Jin-Lan Li

**Affiliations:** 1 Guizhou Center for Disease Control and Prevention, Institute for Tuberculosis Prevention and Control, Guizhou, 550004 China; 2 Haixiao Street Community Health Service Center, Guizhou, 563200 China

**Keywords:** Environmental sciences, Diseases, Medical research

## Abstract

China has implemented a series of long-term measures to control the spread of COVID-19, however, the effects of these measures on other chronic and acute respiratory infectious diseases remain unclear. Tuberculosis (TB) and scarlet fever (SF) serve as representatives of chronic and acute respiratory infectious diseases, respectively. In China’s Guizhou province, an area with a high prevalence of TB and SF, approximately 40,000 TB cases and hundreds of SF cases are reported annually. To assess the impact of COVID-19 prevention and control on TB and SF in Guizhou, the exponential smoothing method was employed to establish a prediction model for analyzing the influence of COVID-19 prevention and control on the number of TB and SF cases. Additionally, spatial aggregation analysis was utilized to describe spatial changes in TB and SF before and after the COVID-19 outbreak. The parameters of the TB and SF prediction models are R^2^ = 0.856, BIC = 10.972 and R^2^ = 0.714, BIC = 5.325, respectively. TB and SF cases declined rapidly at the onset of COVID-19 prevention and control measures, with the number of SF cases decreasing for about 3–6 months and the number of TB cases remaining in decline for 7 months after the 11th month. The spatial aggregation of TB and SF did not change significantly before and after the COVID-19 outbreak but exhibited a marked decrease. These findings suggest that China’s COVID-19 prevention and control measures also reduced the prevalence of TB and SF in Guizhou. These measures may have a long-term positive impact on TB, but a short-term effect on SF. Areas with high TB prevalence may continue to experience a decline due to the implementation of COVID-19 preventive measures in the future.

## Introduction

COVID-19 first emerged in Wuhan city, Hubei province, China, and has since spread to many countries worldwide beginning in early 2020. Cases of COVID-19 were also reported in Guizhou, China^1^. In response to the pandemic, China implemented a series of normalized measures aimed at preventing and controlling the spread of the virus. Some of the critical early measures included requiring people to wear masks in public places, such as buses, subways, hospitals, cinemas, shopping malls, and other crowded locations. Additionally, individuals were advised to reduce participation in crowded activities during the peak of the pandemic, and public places underwent more frequent disinfection. These measures primarily aimed to decrease the likelihood of infected individuals releasing pathogens into the air and reducing the possibility of healthy individuals inhaling pathogens from the environment.

Located in southwest China, Guizhou is an inland region characterized by karst landforms and home to 49 ethnic groups. COVID-19 emerged in the region in early 2020^2^. Starting in February 2020, Guizhou fully implemented a series of national measures to prevent the spread of COVID-19. These measures were in place for over 2 years, and from February 26, 2020, to September 2021, no new local COVID-19 patients emerged in Guizhou under these conditions^3^. Furthermore, Guizhou is an area with high tuberculosis (TB) and scarlet fever (SF) prevalence, reporting approximately 40,000 TB cases and hundreds of SF cases annually^4–7^. Both TB and SF are respiratory infectious diseases^8^, with no currently effective vaccines to prevent them. TB is a chronic respiratory infectious disease caused by Mycobacterium tuberculosis^9^, while SF is an acute respiratory infectious disease caused by hemolytic streptococcus^10^.

In this study, TB and SF were selected as representatives of chronic and acute respiratory infectious diseases, respectively. The aim was to assess whether these respiratory infectious diseases were affected by the normalized COVID-19 prevention and control measures in areas with high TB and SF prevalence and to determine the nature of the impact. This study may provide guidance for TB and SF prevention and control in such areas in the future. By using historical data of TB and SF cases, a prediction model was established to predict the number of TB and SF cases, and then the predicted numbers were compared with the actual numbers in the context of COVID-19. Additionally, the spatial distribution of TB and SF cases was mapped before and after the COVID-19 outbreak.

## Materials and methods

### Date source and study design

The case data for this study were obtained from the China Disease Control and Prevention Information System, while demographic data, such as resident population, were sourced from the Guizhou Provincial Bureau of Statistics. A time series modeler was employed to fit the prediction model, and spatial distribution maps of TB and SF were created for the counties in Guizhou. System parameters for identifying TB and SF patients were set based on onset time, confirmed cases, and the resident population in Guizhou. The SPSS 22.0 software was utilized to establish the prediction model, and the numbers of TB and SF cases in Guizhou were predicted using the exponential smoothing model^11,12^. Case data from January 2010 to June 2019 were used to fit the exponential smoothing model, while data from July 2019 to February 2020 were used to test the forecasting effect. Ultimately, the prediction model was applied to estimate the number of TB and SF cases from February 2020 to June 2021, which were then compared with the actual data^13–16^. The ArcGIS 10.2 software was used to depict the spatial aggregation changes of TB and SF in Guizhou before and after the COVID-19 outbreak^17,18^.

Following the COVID-19 outbreak, aside from prevention and control measures for COVID-19, no other large-scale and more potent prevention and control measures for TB and SF were implemented in Guizhou province until June 2021. Moreover, there was no significant immigration or emigration of the population in Guizhou Province during this period. Furthermore, the COVID-19 epidemic in Guizhou did not impact the resident population, as the number of COVID-19 patients in Guizhou during this period was only 146 with fewer than 10 deaths, hardly affecting the TB and SF populations^19^. Consequently, other major factors affecting TB and SF were excluded from the study design. This made it feasible to investigate the impact of normalized COVID-19 prevention and control measures on TB and SF.

### Time series analysis

The exponential smoothing method is a time series analysis and prediction approach developed based on the moving average method proposed by Robert G. Brown^20^. This method predicts future phenomena by calculating the exponential smoothing coefficient and combining it with a time series prediction model. Generally, more recent data will have a greater impact on the present, while more distant data will have less influence. The fundamental concept of the exponential smoothing method is to consider the effect of time intervals on time development, where the weight of each option decreases exponentially as the time interval increases. The prediction steps of the exponential smoothing method include: (1) plotting a sequence graph; (2) determining effective parameters based on the sequence graph; (3) drawing a fitting curve and observing the fitting effect; (4) establishing an exponential smoothing model to predict data. The exponential smoothing model encompasses three essential parameters, namely conventional parameters, trend parameters, and seasonal parameters. Typically, the overall mean value, overall trend, and seasonality should be utilized for prediction, and various combinations of parameter values should be used for fitting. By comparing the root mean square error (RMSE), mean absolute percent error (MAPE), and mean absolute error (MAE), the optimal model can be selected and the prediction impact of the model can be evaluated.

### Model evaluation

In the model, R^2^ represents the proportion of data variation that can be explained by the model relative to the total variation. The Ljung-Box (18) test is a random test of residual errors in the model, indicating whether the specified model is accurate. A significance of P < 0.05 suggests that the residual error is not random, and there are structures in the observed sequence that the model cannot explain. The optimal prediction model is selected based on an insignificant Ljung-Box (18) test result, maximum R^2^ value, RMSE, MAPE, Bayesian Information Criterion (BIC), and the minimum average relative error of prediction^21^.

### Analysis of spatial aggregation

The Natural Breaks Method was employed to describe the case distribution of TB and SF. To compare the changes in the number of cases in each region before and after the COVID-19 outbreak, the number of TB and SF cases in the spatial distribution map were artificially divided into eight grades, with each grade represented by eight distinct colors.

### Statistic analysis

SPSS 22.0 was used to establish the database, time series diagram, and prediction model, with a test level α = 0.05. ArcGIS 10.2 software was utilized to create the case distribution maps of TB and SF.

## Results

### Construction of prediction model

**Figure 1:**
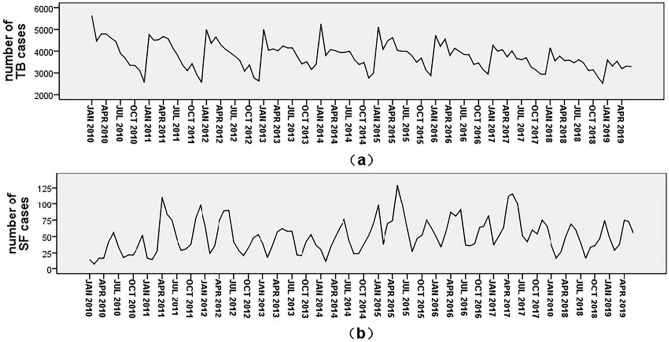
Time series of TB (**a**) and SF (**b**) cases from January 2010 to June 2019.

**Figure 2:**
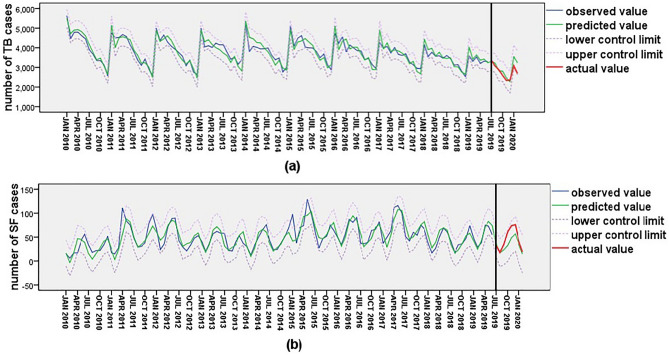
The forecasting effect of TB (**a**)and SF (**b**)prediction models.

The time series diagram of Guizhou’s TB and SF cases from January 2010 to June 2019 revealed some regularities in their onset times (Fig. 1). The number of TB cases peaked in January (Fig. 1a), while the number of SF cases peaked in May to June and November to December (Fig. 1b). Utilizing these data and accounting for the regularities to fit the exponential smoothing model, the model parameters for TB were R^2^ = 0.856, RMEC = 231.47, MAPE = 4.924, and Standard BIC = 10.972, while the exponential smoothing model parameters for SF were R^2^ = 0.714, RMEC = 13.75, MAPE = 23.465, and Standard BIC = 5.325 (Table 1). Furthermore, the data from July 2019 to February 2020 were used to test the forecasting effects of the two models, which demonstrated excellent prediction effects, with actual values being highly consistent with predicted values and falling between the upper and lower limits of the 95% CI (Fig. 2a,b). The prediction model parameters and forecasting effects indicated that the two prediction models were successful.

**Table 1:** Parameters of exponential smoothing models for TB and SF.

Disease	*R*^2^	RMEC	MAPE	Standard BIC	Ljung-BoxQ (18)	
					Statistics	*P* value
Tuberculosis	0.865	231.47	4.924	10.972	21.181	0.172
Scarlet fever	0.714	13.75	23.465	5.325	21.151	0.173

### Changes of TB and SF cases number under the background of COVID-19 prevention and control

**Figure 3:**
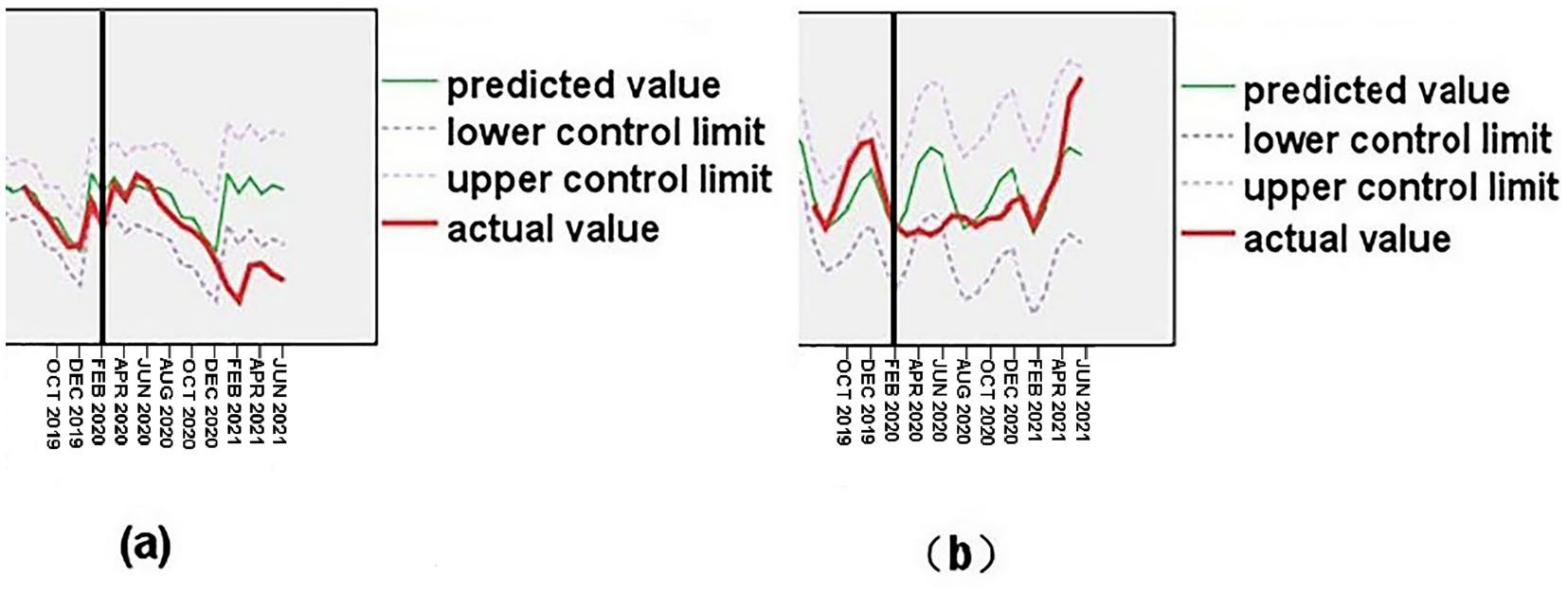
Changes in TB (**a**) and SF (**b**) number under under the background of COVID-19 prevention and control.

Guizhou province initiated normalized prevention and control measures to manage COVID-19 starting in February 2020. The successful prediction model was utilized to estimate the number of TB and SF cases in Guizhou province from February 2020 to June 2021. The actual number of TB cases declined compared to the predicted value from February to March 2020 but remained within the predicted value range until November 2020. From December 2020 until June 2021, the actual value started to deviate from the predicted value, with the actual number of cases per month being lower than the predicted value (Fig. 3a). The actual value of SF cases was significantly lower than the predicted value from February 2020 to July, exhibiting a trend of initially decreasing and then slowly increasing. This trend contrasted with the curve shape of the predicted number of SF cases, and the actual number of SF cases from April to June 2020 was lower than the predicted value range. The actual curve of SF gradually returned to the prediction curve starting in August 2020 (Fig. 3b).

### Analysis of spatial aggregation

**Figure 4:**
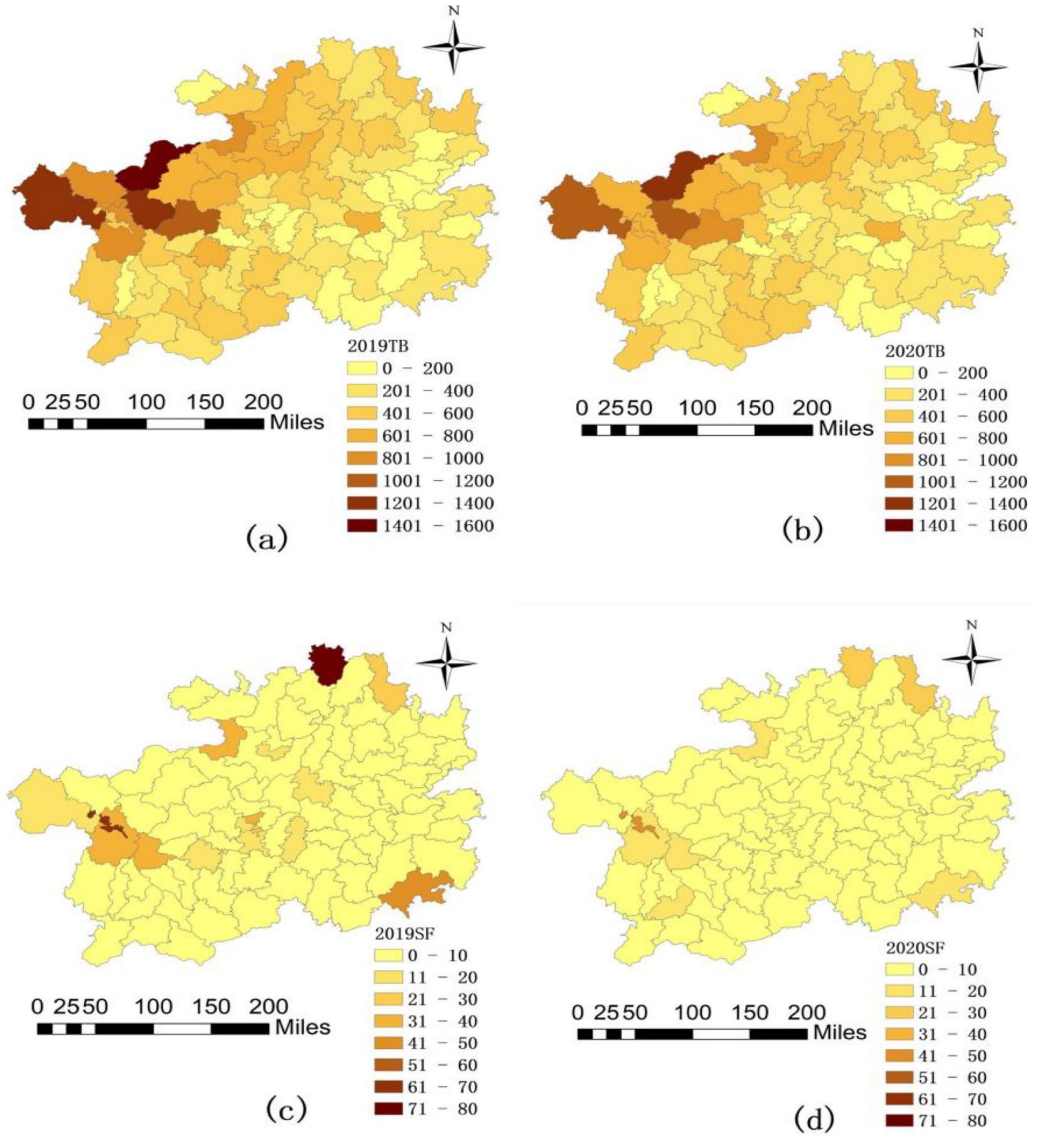
Spatial distribution of TB and SF cases before and after COVID-19 in Guizhou, (**a**) 2019 TB, (**b**)2020 TB, (**c**) 2019SF, (**d**) 2020SF. (Map version number: Qian S(2020)007, URL link: https://maifile.cn/est/d3156790612696/pdf).

In 2019, the number of TB cases in Guizhou province reached 36,286, while in 2020, it decreased to 34,384. TB cases declined in all counties in 2020 compared to 2019, with some counties that had more than 1000 TB cases in 2019 experiencing significant decreases in 2020. High TB prevalence remains concentrated in western Guizhou (Fig. 4a,b).

In 2019, Guizhou province reported 616 SF cases, which dropped to 278 in 2020. SF cases diminished in all counties in 2020 compared to 2019. The counties with 31–40, 41–50, 61–70, and 71–80 SF cases in 2019 witnessed significant reductions in 2020 (Fig. 4c,d).

## Discussion

Guizhou province, situated in southwest China, has a high incidence of TB and SF. The COVID-19 pandemic swept the world in 2020, prompting Guizhou to implement a series of prevention and control measures. Among these, the most critical measure mandated wearing masks in public places, such as buses, subways, hospitals, cinemas, and shopping malls^22,23^. People were also advised to limit participation in crowded activities during the worst of the pandemic, and public gathering places were disinfected more frequently than before^24,25^. The primary purpose of these actions was to reduce the likelihood of COVID-19 patients releasing pathogens into the air and healthy individuals inhaling them. These prevention and control measures were in place in Guizhou province until September 2021, with no new local COVID-19 patients reported^26,27^. This study aims to explore the impact of long-term COVID-19 prevention and control on other respiratory infectious diseases, selecting TB and SF as representatives of chronic and acute respiratory infectious diseases, respectively.

The study utilized the exponential smoothing model to predict the number of TB and SF cases in Guizhou Province, with the parameters and test results of the prediction model demonstrating its success. The research revealed that COVID-19 prevention and control measures also positively impacted TB and SF, suggesting similar effects on other respiratory infectious diseases. The most useful measure was wearing a mask^28,29^, and the study's results indicated varying impacts on acute and chronic respiratory infectious diseases. Some research has demonstrated that the COVID-19 pandemic affected the number of infectious disease cases based on their mode of transmission^30,31^. However, these studies only showed a decline in some infectious diseases due to the pandemic, rather than using predictive models to explore the causes, such as the duration and intensity of the impact on different infectious diseases.

A rapid decline in both TB and SF cases occurred in the first 1–2 months under COVID-19 prevention and control measures. At that time, the focus was primarily on COVID-19, with active detection of other infectious diseases, such as TB and SF, being reduced or temporarily halted. Furthermore, patients' willingness and behavior in seeking medical care were affected. Additionally, the COVID-19 prevention and control measures reduced the spread of SF, an acute respiratory infectious disease with an incubation period as short as 1–7 days^32,33^. This led to a short-term decrease in SF cases.

Moreover, the research results showed that the long-term decrease in the number of SF cases did not persist in the context of COVID-19 prevention and control measures, returning to the predicted state about 7 months later. This was likely due to the public's declining willingness to comply with regular prevention and control measures and increasing psychological fatigue^34^. In contrast, apart from the initial 1–2 month decline, TB began to decline significantly after the 11th month, with the downward trend lasting 7 months or even longer. As TB is a chronic respiratory infectious disease with a long incubation period (from 6 months to over a year or more)^35,36^, COVID-19 prevention and control measures reduced the spread of Mycobacterium tuberculosis and infection possibilities in healthy people. However, the effect could only be observed after several months, becoming particularly evident in areas with severe TB epidemics, such as Guizhou province.

The study revealed that the normalized COVID-19 prevention and control measures in Guizhou province might have a long-term impact on TB, potentially maintaining a decline in the number of TB cases for 7 months or even longer after 11 months of implementation. In contrast, the impact of these measures on SF may be short-term, with cases declining for approximately 6 months. It is also believed that limited contact between people due to lockdowns, working from home, and stay-at-home strategies played a crucial role in decreasing exposure to pathogens related to diseases such as TB and SF.

Spatial aggregation analysis demonstrated that the normalized COVID-19 prevention and control measures effectively reduced TB and SF cases. In 88 counties in Guizhou, TB and SF cases exhibited reductions to varying degrees, particularly in areas with the highest number of cases. However, these areas remain the most severely affected.

## Conclusions

During the COVID-19 pandemic in China, the public was required to wear masks in gathering places, reduce outdoor gatherings, and ensure that gathering places were regularly disinfected and sterilized. These long-term preventive measures proved highly effective in the prevention and control of respiratory infectious diseases, not only successfully preventing COVID-19 but also significantly reducing the number of TB and SF cases. These prevention and control measures may lead to a long-term positive impact on TB and a short-term impact on SF, providing crucial guidance for future TB prevention and control efforts in China, particularly in areas with high TB incidence.

## Data Availability

The data used in this study are freely available. All data generated and analysed during the course of this study are available from the corresponding author upon request. (Data link: https://maifile.cn/est/d2356790650829/pdf).
